# RETRACTION: The REDD1/TXNIP Complex Accelerates Oxidative Stress‐Induced Apoptosis of Nucleus Pulposus Cells through the Mitochondrial Pathway

**DOI:** 10.1155/omcl/9767849

**Published:** 2026-05-22

**Authors:** Oxidative Medicine and Cellular Longevity

RETRACTION: H. Yin, K. Wang, A. Das, et al., “The REDD1/TXNIP Complex Accelerates Oxidative Stress‐Induced Apoptosis of Nucleus Pulposus Cells through the Mitochondrial Pathway,” *Oxidative Medicine and Cellular Longevity*, 2021, 7397516, https://doi.org/10.1155/2021/7397516.

The above article, published online on 22 September 2021 in Wiley Online Library (wileyonlinelibrary.com), has been retracted by agreement between the Journal Editor‐in‐Chief, Jeannette Vasquez‐Vivar; and John Wiley & Sons Ltd. The retraction has been agreed due to multiple concerns identified with the figures, initially on PubPeer [1]. Further investigation identified additional inconsistencies. Please find a summary of the concerns below:

• In Figure 1a, the third and fourth REDD1 bands appear highly similar to the TXNIP bands in Figure 4a
• In Figure 4b, the first three TXNIP bands appear highly similar to the TXNIP bands shown in Figure 6d, after being flipped vertically
• In Figure 4b, the first three GAPDH bands shown appear highly similar to the GAPDH bands shown in Figure 6d, after being flipped vertically
• The first two plots shown in Figure 5b appear to be identical
• In Figure 6a, the p53 bands appear highly similar to the REDD1 blots in Figure 7c

After assessment of the concerns and the author’s responses by the Editorial Board, the results and conclusions can no longer be considered reliable, and the article is retracted.

Cao Yang, Huipeng Yin, Gaocai Li, Shuai Li, Yu Song and Rongjie Luo agree to the retraction. The remaining authors were unresponsive.
